# On the Oleate of Mercury

**Published:** 1873-08

**Authors:** 


### On the Oleate of Mercury.

Mr. Berkeley Hill states (*Practitioner*, April, 1873), that since this preparation was introduced to the notice of the profession by Mr. Marshall, about a year ago, I have employed it in a large number of cases in hospital and private practice, with the following results: In the first place, if continuously applied, it quickly produces the usual effects of mercury on the system, and if used in sufficient quantity causes salivation. Secondly, it is apt, in delicate fair-skinned persons, to excite violent smarting pain, which, though rarely lasting more than half an hour, if so much, is enough to disgust them with the remedy. The irritation may even cause erythema and slight vesication, though I have never seen any more serious local effect than this. To avoid these undesirable occurrences, Mr. Marshall has devised three preparations of different strengths, containing 20, 10 and 5 per cent. of peroxide of mercury respectively: to the weakest dilution, 10 per cent. of morphia as oleate of that base is added, to allay the irritation from the mercury, and assuage the local pain of inflammation, when used for affections of that kind.

The preparations are best made according to a formula prescribed by Mr. Martindale, the dispenser to University College Hospital: For the 20 per cent. solution, stir 10 drachms of oleic acid in a mortar, while 2 drachms of precipitated peroxide of mercury are gradually sprinkled into it, and triturate frequently during twenty-four hours, until the peroxide is dissolved and a gelatinous solution is formed. The 10 per cent. solution is made in exactly the same way, but the smaller quantity of oxide renders the compound more fluid. The morphia and mercury oleate is made by dissolving one drachm of pure alkaloid of morphia in 5 drachms of oleic acid and mixing the solution with 5 drachms of 10 per cent. oleate of mercury. It is necessary to use the oxide freshly precipitated from an aqueous solution, not one produced by dry heat; and heat should not be employed to dissolve the mercury in the acid, as even very moderate elevation of temperature causes some decomposition of the oxide to take place.

With one or other of these preparations the application of this form of mercury can be continued on even very sensitive skins. When used for inunction, instead of the grey ointment, about a scruple or half a drachm of the 20 per cent. jelly should be rubbed gently into the flank till it is absorbed by the skin, which occurs in about eight or ten minutes, leaving the skin almost dry and not greasy. This may be repeated once or twice in twenty-four hours, of course changing the sight of the inuction each time. The anointed part may be washed next day without fear. This quantity usually causes swelling and slight soreness of the gums in a week, if anointed once a day, and in four days if applied twice daily. Before using the stronger solution it is well to test the skin with the weaker form, lest too energetic application of the oleate should cause painful irritation and trouble. But I have found the 10 per cent. solution most useful as an adjuvant to the ordinary treatment by iodide of potash internally, or for persons whose stomachs do not bear mercury well. For example, in cases of leproid, or tubercular eruptions, relapsing after disappearing more than once, this form of mixed treatment is usually very successful.

The great advantage of the oleate over any other form of mercury, when externally applied, lies in the rapidity of its absorption, which makes it very serviceable as a kind of cosmetic; that is, to paint over syphilitic papules or stains in the face or other exposed parts. For this purpose I direct the patient to rub into the spots themselves, night and morning, a little of the 20 per cent. solution with the tip of the finger—the usual treatment being continued, at the same time. It is remarkable to observe how rapidly the papules sink down and grow pale when the oleate is directly applied to them. If the 20 per cent. is too stimulating, the weaker ones may be employed, though their effect is less satisfactory.

Again, the oleates are very useful in fissures of the fingers about the nails or in the palms. Rubbing the 10 per cent., or, if there is much soreness, the 5 per cent. solution with morphia, into the fingers, at night, and sleeping in wash-leather gloves, is a very effectual way of healing these troublesome affections. By day the cracks should be well closed by court-plaster and plastic collodion, and gloves worn out of doors.

I have not had much success with the oleate in non-syphilitic affections, but I have not tried it extensively. It has proved a very effective parasiticide for pediculi, as its penetrating power enables it to diffuse itself thoroughly over the scalp and pubis. I have also used it to inflamed joints, as a controllant of inflammatory action, but I have not perceived any clear benefit to be derived from its use in such cases. In syphilitic affections the oleate is most serviceable, being a certain and less disagreeable cutaneous application than ointments, and really hastening the subsidence of papules and other disfigurements of exposed parts of the skin.—*American Journal Med. Sciences.*

